# Effect of AT1 receptor blockade on cardiovascular outcome after cardiac arrest: an experimental study in rats

**DOI:** 10.1038/s41598-023-45568-4

**Published:** 2023-10-25

**Authors:** E. A. F. Araújo Filho, M. J. C. Carmona, D. A. Otsuki, D. R. R. Maia, L. G. C. A Lima, M. F. Vane

**Affiliations:** 1 Departamento de Cirurgia (LIM 08), Faculdade de Medicina da USP (FMUSP), EAF: Av. Dr. Arnaldo, 455, sala 2120 (LIM-08), São Paulo, SP 01246-903 Brazil; 2 Departamento de Cirurgia, Disciplina de Anestesiologia, Universidade de São Paulo, São Paulo, Brazil; 3 Departamento de Patologia, Faculdade de Medicina da USP (FMUSP), São Paulo, Brazil

**Keywords:** Microbiology, Neuroscience, Physiology, Cardiology, Neurology

## Abstract

Angiotensin II receptor 1(AT1) antagonists are beneficial in focal ischemia/reperfusion (I/R). However, in cases of global I/R, such as cardiac arrest (CA), AT1 blocker's potential benefits are still unknown. Wistar male rats were allocated into four groups: Control group (CG)—animals submitted to CA by ventricular fibrillation induced by direct electrical stimulation for 3 min, and anoxia for 5 min; Group AT1 (GAT1)—animals subjected to CA and treated with 0.2 mg/kg of candesartan diluted in dimethylsulfoxide (DMSO) (0.1%); Vehicle Group (VG): animals subjected to CA and treated with 0.2 ml/kg of DMSO and Sham group (SG)—animals submitted to surgical interventions, without CA. Cardiopulmonary resuscitation consisted of group medications, chest compressions, ventilation, epinephrine (20 mcg/kg) and defibrillation. The animals were observed up to 4 h after spontaneous circulation (ROSC) return, and survival rates, hemodynamic variables, histopathology, and markers of tissue injury were analyzed. GAT1 group had a higher rate of ROSC (62.5% vs. 42.1%, *p* < 0.0001), survival (100% vs. 62.5%, *p* = 0.027), lower incidence of arrhythmia after 10 min of ROSC (10% vs. 62.5%, *p* = 0.000), and lower neuronal and cardiac injury scores on histology evaluation (*p* = 0.025 and *p* = 0.0052, respectively) than GC group. The groups did not differ regarding CA duration, number of adrenaline doses, or number of defibrillations. AT1 receptor blockade with candesartan yielded higher rates of ROSC and survival, in addition to neuronal and myocardial protection.

## Introduction

Cardiac arrest (CA) is the most severe adverse event in the perioperative scenario. Even if promptly assisted, it has a high mortality rate, ranging from 35% immediately after the event to approximately 70% after one year^1–4^. Several studies have discussed ways to intervene and reduce the damage caused by global ischemia due to CA^5–7^.

Recent studies have demonstrated the use of renin-angiotensin system (RAS) inhibitors as attenuators of tissue damage in ischemia–reperfusion (I/R)^8–12^. Angiotensin II, the main active principle of the RAS, exerts its effect primarily by stimulating angiotensin receptors 1 (AT1R), specifically in the brain, AT1R are highly expressed in cerebrovascular endothelial cells and have an essential role in regulating cerebrovascular flow and the function of the blood–brain barrier^13, 14^. Angiotensin II has also been linked to direct neurotoxic effects, leading to intracellular generation of reactive oxygen species, increased production of inflammatory cytokines and downregulation of the anti-inflammatory peroxisomes^13, 15, 16^. Thus, angiotensin receptor antagonists (ARA) have been shown to protect cerebral blood flow, maintain blood–brain barrier function, prevent excessive brain inflammation and neuronal injury in animal models of stroke, traumatic brain injury, Alzheimer's and Parkinson's disease. Direct neuroprotection has also been demonstrated, for candesartan and telmisartan, attenuating known mechanisms of neuronal damage by reducing glutamate excitotoxicity, excessive pro-inflammatory interleukin-1beta (IL-1β), and hypoxia induced-lesions^17–20^. Blockade of the AT1 receptor also provided cardioprotective effects in the setting of ischemia–reperfusion, and has been shown to reduce infarct size, ischemia-related arrhythmias, and attenuate ventricular dilatation after myocardial infarction^21–24^. Specifically, candesartan has been shown to improve the functional recovery of reperfused myocardium, decreasing myocardial stunning^25^.

Although previous studies have shown the benefits of ARA use under localized I/R, there are no studies with the use of ARA in situations of multiple organ ischemia, such as CRA. Therefore, we aimed to evaluate the role of angiotensin II AT1-receptor blockers on myocardial and brain lesions after a CRA, as well as return of spontaneous circulation rates (ROSC) and survival.

## Methods

An experimental study was conducted with male Wistar rats weighing between 380 and 465 g from the Central Animal Facility of the Faculty of Medicine of the University of São Paulo. The Ethics Committee approved the study for the Use of Animals of the University of São Paulo—CEUA-USP, protocol number: 034/16. All experiments were performed in accordance with relevant guidelines and regulations.

After being anesthetized and subjected to surgical procedures, the rats were allocated in a simple random fashion into four different groups.

Sham Group (SH): Anesthetized animals submitted to surgical instrumentation procedures for hemodynamic monitoring but with no ventricular fibrillation (VF) induction.

Control Group (CG): Animals submitted to CRA within 6 min of anoxia, followed by cardiopulmonary resuscitation (CPR). At the start of CPR, 20 mcg/kg of adrenaline and 0.5 ml of 0.9% saline were administered.

Vehicle group (VG): Animals submitted to CRA within 6 min of anoxia, followed by CPR. Adrenaline (20 mcg/kg) and 0.2 ml/kg of 99% dimethylsulfoxide (DMSO) were administered at the beginning of CPR.

Group AT1 (GAT1): Animals submitted to CRA within 6 min of anoxia, followed by CPR. At the beginning of CPR, adrenaline (20 mcg/kg), together with an intravenous dose of the candesartan cilexetil metabolite, Candesartan (> 98%, CAS 139481-59-7), diluted in DMSO (99%) in 0.1%, at a dose of 0.2 mg/kg was injected.

### Induction of experimental cardiorespiratory arrest and resuscitation

After induction of general anesthesia with 1,2% isoflurane (1 MAC), electrocardiographic monitoring and orotracheal intubation were performed with a 16 G flexible venous catheter (B. Braun). Afterward, the animals were subjected to mechanical ventilation with a tidal volume of 8 ml/kg and a respiratory rate of 60 breaths per minute (bpm). A rectal thermometer adjusted body temperature to 37 + /− 1 °C. An incision in the cervical region was performed, and the right internal jugular vein was located. The tip of a 3F polyurethane pediatric venous catheter, size 0.6 mm ID and 1.0 mm OD (3F), was cut at a 45° angle for advancement into the right atrium.

The 3F external jugular catheter was marked 4 cm from the tip. This same catheter was used to advance a guidewire into the right ventricle for electrical induction of VF with the subsequent option of using it for drug delivery and blood sampling. Next, the inguinal region was incised, and another PE10 catheter was introduced into the right common femoral artery to collect parameters and invasively measure blood pressure using a data acquisition system (MP-100, BIOPAC, USA). The electrocardiographic (ECG) analysis was performed using a module coupled to the same system.

For CPR induction, the catheter was inserted through the jugular and guided by the pressure curve to the right ventricle. Through this, an electrical conductor was introduced, and an electrical discharge of 1 mA (9 V, 60 Hz) was applied and maintained for 3 min. Simultaneously, mechanical ventilation was discontinued. Then, the guide was removed, and the venous catheter was retracted 1 cm and fixed for drug administration. The animals were maintained for another 3 min in CPR, totaling 6 min from the beginning of VF induction. Confirmation of VF induction was confirmed by observing the cessation of aortic pulsations, an exponential drop in aortic pressure below 20 mm Hg and the appearance of disorganized electrical activity on the ECG. After, cardiopulmonary resuscitation maneuvers were started. An automatic compressor performed external chest compressions at a frequency of 200 compressions per minute and chest depth of 1.5 cm^26^. Together with chest compression, ventilation was reestablished (RR-25ipm), and a mixture of adrenaline (20mcg/kg) and sodium bicarbonate (1 mEq/kg) was administered.

**Figure 1:**
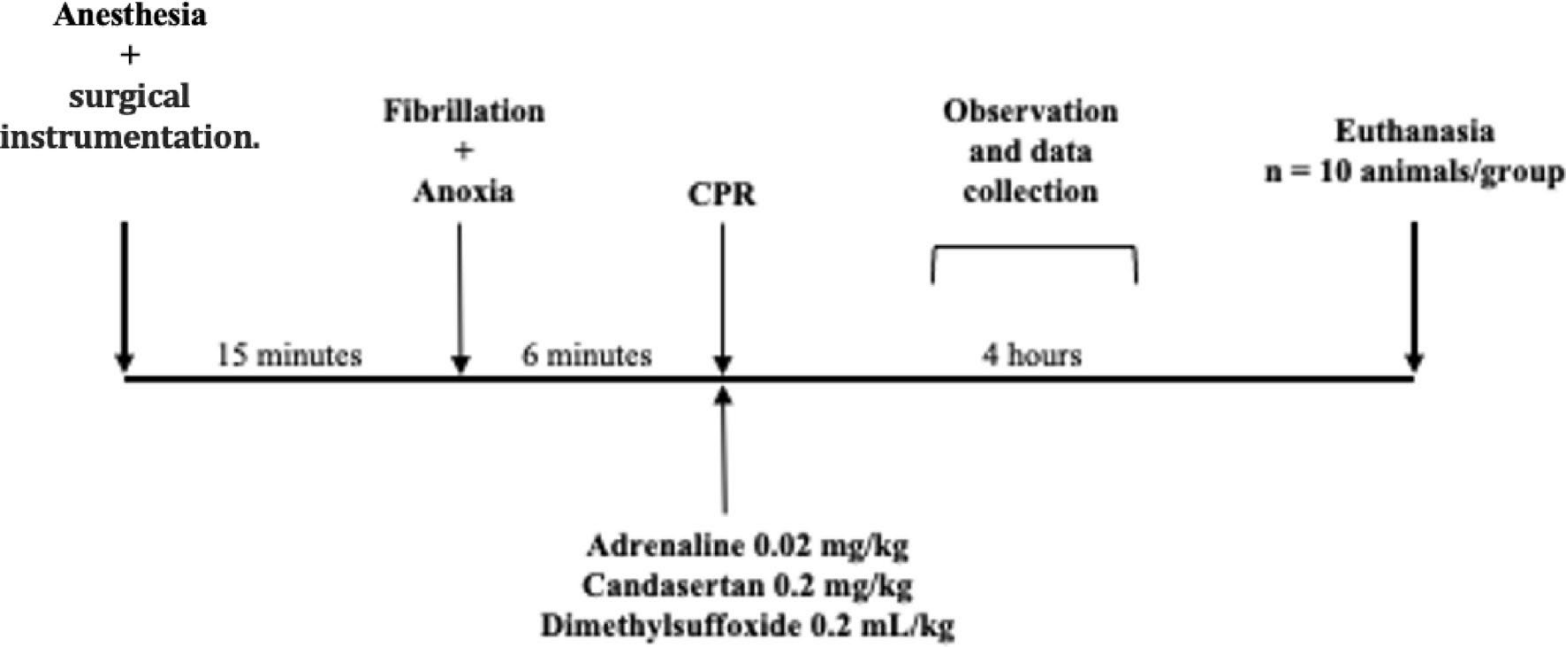
Study protocol. CPR: cardiopulmonary resuscitation. Surgical instrumentation: invasive arterial pressure, venous central access.

The heart rate was checked every 3 min. If VF was present, a 7 J discharge was performed. After defibrillation, CPR was resumed for another 3 min, together with a new dose of adrenaline. After a heart rate check, arterial pulsations were taken as a ROSC. The maintenance of mean arterial pressure above 25 mmHg for 10 min was considered sustained ROSC as described in the protocol by Lamoureux et al., 2015^27^. If there were no pulsations in the second check and still in VF, a new defibrillation load of 7 J was applied, and chest compressions were resumed, adding a fresh dose of adrenaline. Then, new defibrillations and rhythm checks were performed every 2 min, and adrenaline was administered every 3 min. After 20 min of CPR, efforts were finalized. However, if sustained ROSC and hemodynamic stability were established, the animals were observed for another 4 h (Fig. 1).

The animals surviving the 4-h period had a blood sample collected for troponin measurement. After, they were sacrificed by an overdose of inhalational anesthetic (6% isoflurane). Afterward, the heart and brain were extracted for processing and stored partly in formalin and partially frozen at − 70 °C.

### Outcomes assessment

#### Hemodynamic evaluation

Blood pressure, heart rate, and cardiac rhythm were continuously recorded from the beginning of surgical instrumentation procedures, after anesthetic induction, and until animal sacrifice. The parameters were recorded on data sheets every 10 min, from the ROSC until the end of the experiment.

To analyze the quality of CPR maneuvers, diastolic blood pressure was observed for values above 20 mmHg. Cardiac arrhythmias, when present, were classified as tachyarrhythmia, bradyarrhythmia, ventricular arrhythmia, or atrial arrhythmia, according to the Lambeth Convention guideline^28^.

#### Laboratorial determination

Blood samples (1 mL in a heparinized syringe) were collected at pre-CPR induction, after CPR, and after 4 h of observation. Values for pH, pO_2_, pCO_2_, lactate, electrolytes (sodium, potassium, chlorine, and calcium), hemoglobin, hematocrit and troponin I were checked. Samples were allocated in specific tubes and analyzed by an ABL800 Flex Radiometer device**.**

#### Histology

After the animals were sacrificed, the brain and heart were carefully dissected and immediately fixed in 4% paraformaldehyde in phosphate buffer pH 7.0 for 24 h. After, they were dehydrated in an alcoholic gradient (70º to 100º), cleared in xylol, and embedded in paraffin. 5 µm cuts were obtained for morphological and morphometric evaluation.

The evaluation was performed using tissue morphometry and the percentage of inflammatory cells per unit area of parenchyma. In each histological section, digital images of 10 random fields were obtained, covering the full extension of the tissue, using the Image Pro-Plus program and a light microscope at 400 × magnification. For the analysis, we used a programming tool to calculate the area occupied by the parenchyma.

The pathologist responsible for the analysis was blind to the animal group and the corresponding slide. The histopathological evaluation of both the brain and the myocardium was scored based on the percentage of lesions through manual quantification for each photomicrograph: 1 = (< 30%); score 2 = (31%–60%); score 3 = (> 60%)^29^.

### TUNEL

The Terminal deoxynucleotidyl transferase-mediated (dUTP) nick end Labeling kit, also called the Tunel labeling index, was used to analyze cell injury and apoptosis. Tissue section 4–5 µm thick were placed on silanized slides (Sigma Chemical Co.; St. Louis, Missouri, USA) in a suitable holder.

Using a light microscope (BLUE1600BAL-BAT-Biofocus), the slides were manually counted at 100× magnification. Twenty-five brain fields and 50 heart fields were analyzed. For the heart, 25 were from the right ventricle, and 25 were from the left. Means for each slide and the median for each group were then calculated. The slides were blindly read for each group.

### Statistical analysis

The sample calculation was obtained from the troponin value after cardiac arrest in previous studies^30, 31^. Nine animals per group were needed for a 15% difference between groups, with a type 1 error (α) of 0.05 and a power of 0.80 for a two-tailed test. However, anticipating losses, 10 animals per group were estimated. As the current PCR model presents a mortality of 50% of the animals, the availability of 64 animals for the study was suggested. Initially, descriptive measures were obtained for the minimum and maximum values, mean, and standard deviation variables. Such descriptive measures discriminated the data according to the study period (post and final) and according to the groups.

All continuous variables are presented as Mean ± SD after the Kolmogorov‒Smirnov test confirms the normality. Data were analyzed by one-way analysis of variance (1-way ANOVA) or 2-way ANOVA, where appropriate, followed by post hoc Tukey tests for comparisons between different groups. Non-normal data were analyzed by Kruskal–Wallis. Fisher's exact test (chi-square) was used to analyze CPR rate, survival, and myocardial and brain injury scores. Statistical significance was assumed for *p* < 0.05. Statistical analyzes were performed in SPSS version 23.0 for Windows (SPSS, Chicago, IL).

### Ethical approval

An experimental study was carried out with rats, set in the Laboratory of Medical Investigation of Anesthesiology (LIM-08), at the Faculty of Medicine of the University of São Paulo, approved by the Commission for Ethics in the Use of Animals of the University of São Paulo (CEUA- USP), protocol number: 034/16.

## Results

**Figure 2:**
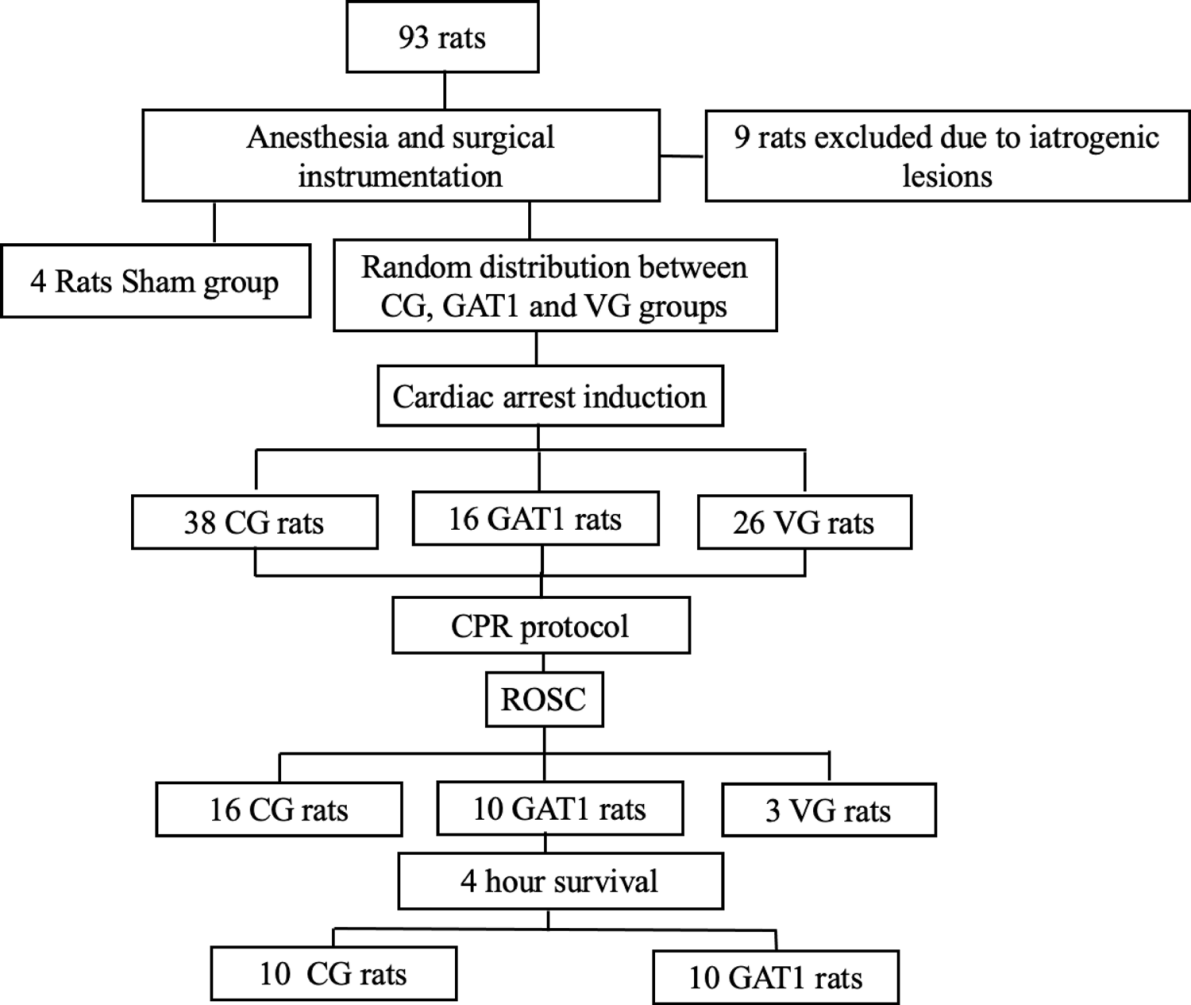
Study flow diagram. CG: Control group; GAT1: Candesartan group; VG: Vehicle group; CPR: Cardiopulmonary resuscitation; ROSC: Return of Spontaneous Circulation.

The study was conducted with 93 rats to achieve the sample size of 10 animals surviving 4 h after ROSC in GAT1 and GC. The VG was excluded from the analysis, since among the 26 animals used in the vehicle group, only 3 met ROSC criteria, and none completed the observational period (Fig. 2). Nine animals were discarded (five due to iatrogenic injury and four due to failure to induce CPR).

## Baseline and cardiopulmonary resuscitation parameters

There were no significant differences in baseline physiological and hemodynamic parameters among the groups before the CRA (Table 1). In addition, there were no differences in CPR duration, the number of adrenaline doses, or defibrillations (Table 2).

**Table 1:** Basal characteristics of the animals.

Variable	SG (n = 4)	CG (n = 38)	GAT1 (n = 16)	p-value
Weight (g)	465.4 ± 38.5	379.5 ± 47.4	409.7 ± 2.87	0.084
Temperature (ºC)	35.7 ± 0.17	35.5 ± 0.8	35.5 ± 0.94	0.985
Heart rate (bpm)	199.5 ± 51.3	193.6 ± 15.6	201.7 ± 43.8	0.942
MAP (mmHg)	76.2 ± 12	93.6 ± 14.8	96.2 ± 12.9	0.290

Data are presented as mean ± SD. One-way ANOVA, p > 0.05; GAT1: Candesartan group; CG: control group; SG: sham group; MAP: mean arterial pressure.

**Table 2:** Cardiopulmonary resuscitation data.

Variable	ROSC		*p*-value	4 h Survival		*p*-value
	CG (n = 16)	GAT1 (n = 10)		CG (n = 16)	GAT1 (n = 10)	
CPR time(min)	4.5 [3; 7]	4.0 [3; 6]	0.888	4.5 [3; 6]	4.0 [3; 6]	0.904
Adrenaline doses	1.5 [1; 2.3]	1.5 [1; 2]	0.753	1.5 [1; 2]	1.5 [1; 2]	0.836
Number of defibrillations	1.0 [0; 1.3]	1.0 [0.3; 1]	0.668	1.0 [0; 0.3]	1.0 [0.3; 1]	0.736

Data are presented as median [1st; 3rd quartiles]. Mann–Whitney test; GAT1: Candesartan group; CG: control group; SG: sham group; MAP: mean arterial pressure;

### Return of spontaneous circulation

The GAT1 had a ROSC rate of 62.5% (10/16), higher than the control group (42.1% (16/38; X^2^: 42.9; *p* = 0.012). Five animals in the control group had a non-shockable rhythm after starting the CPR maneuvers and were not submitted to defibrillation. Three animals in the GAT1 group had a non-shockable rhythm (*p* = 0.94).

**Figure 3:**
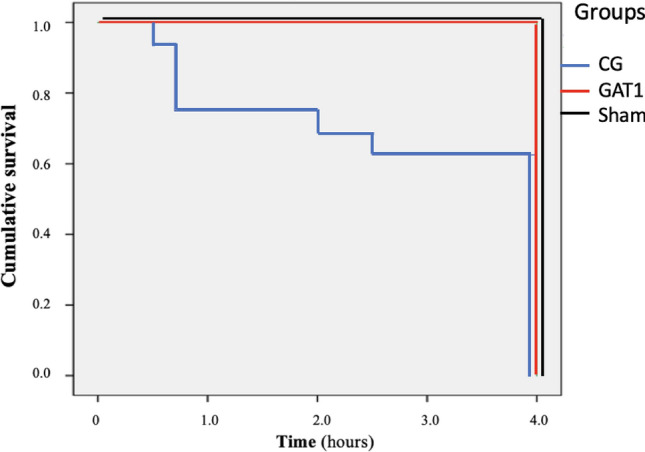
Cumulative survival curve of rats subjected to cardiac arrest and treated with adrenaline (GC, n = 16) or candesartan (GAT1, n = 10) and sham-operated rats (Sham, n = 4).

All animals in the GAT1 that met ROSC criteria survived until the end of the study. Ten (62.5%) of the 16 animals in the control group survived until the end of the established period (Fig. 3) (X^2^: 4 0.87, *p* = 0.027).

## Post-cardiopulmonary resuscitation data

### Hemodynamic variables

**Figure 4:**
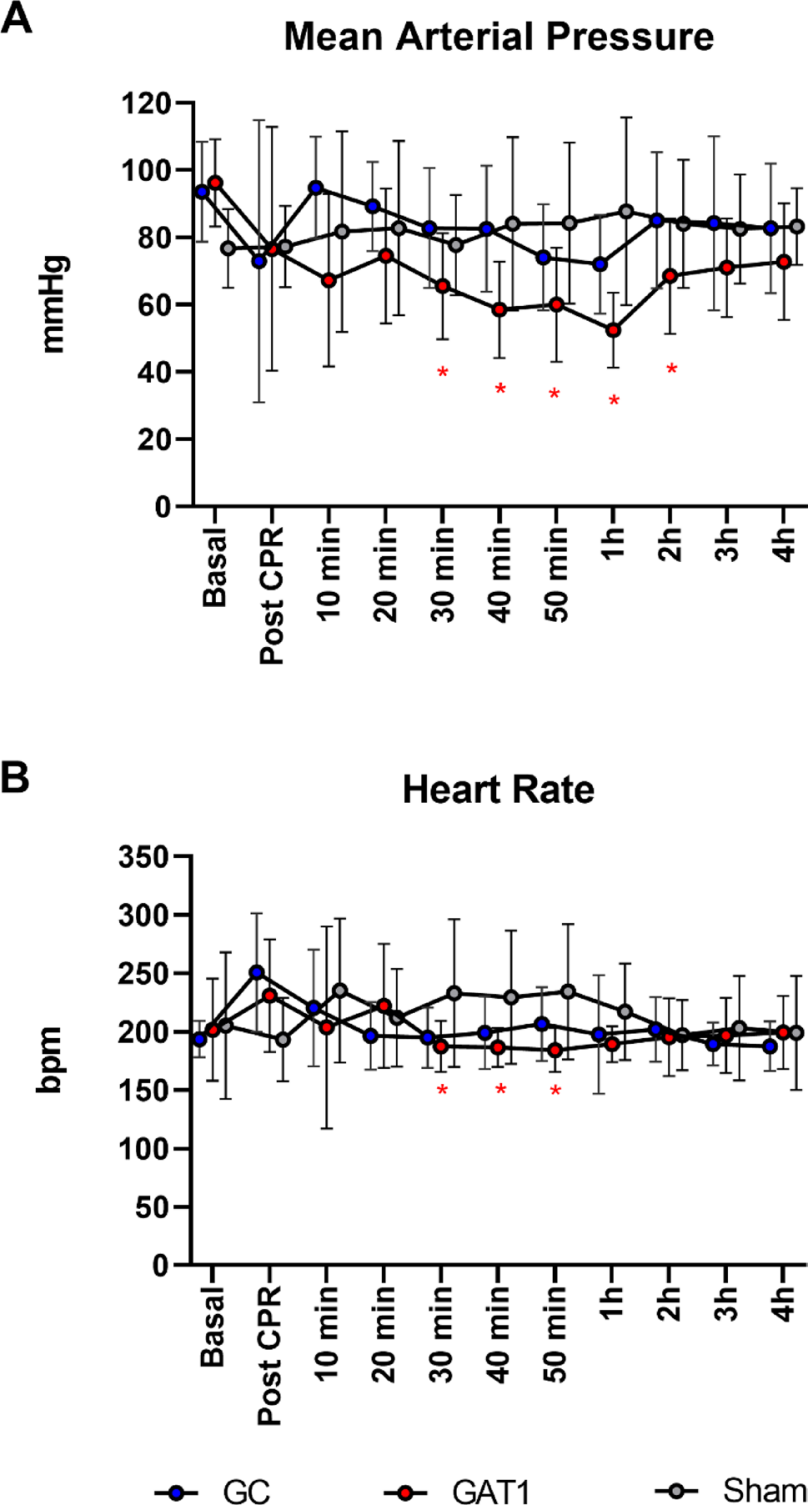
Mean arterial pressure (**A**) and Heart rate (**B**) of rats subjected to cardiac arrest and treated with adrenaline (GC, n = 10) or candesartan (GAT1, n = 10) and sham-operated rats (Sham, n = 4). *p < 0.05 vs GC and Sham.

A significant reduction in blood pressure was observed in the GAT1 group, from 30 min onwards, for the animals that survived during the four hours of the experimental period compared to the GC and Sham groups. The difference was statistically significant up to two hours (*p* = 0,041; Fig. 4A).

The animals in the CG group had more cardiac arrhythmias in the ten-minute interval after ROSC. Eleven animals had arrhythmias, ten from the CG group. In these, non-sustained ventricular tachycardia (NSVT) was observed in seven animals; three had supraventricular arrhythmia. One animal from the GAT1 group had NSVT. This data was statistically significant between groups (X^2^: 4.85; *p* = 0.013). The animals in the Sham group did not have arrhythmias during the observation period. Analyzing the temporal heart rate, it was observed that the GAT1 group had lower median HR than the CG and Sham groups, statistically significant only between 30 and 50 min (CG vs GAT1 *p* = 0.037; 0.044; Fig. 4B).

### Troponin I

The measurement of troponin I was performed 4 h after animal resuscitation. CG group mean was 18,265 ± 8725 pg/mL, while the GAT1 group mean was 18,027 ± 8658 pg/mL, which is not statistically significant. Compared to the Sham group, these values were markedly higher (824 ± 1149 pg/mL).

The animals that met the ROSC criteria showed metabolic acidosis in both groups undergoing CPR. However, the GAT1 group had a higher mean pH than the control group (7.183; 7.101, U: 432, *p* = 0.032). Similarly, higher values were observed for potassium (3.1; 3.35; U: 456 *p* = 0.045), base excess (− 9.75; − 12.1; U: 434 *p* = 0.048), and bicarbonate (17.4; 15.95, U: 509, *p* = 0.037). Data from arterial blood gas at the end of the study protocol was similar between the variables, and acidosis regulation was observed, moving towards standard values over time. Only the hematocrit of the Sham group was lower than the other groups. Medians for lactate, base excess, and bicarbonate showed no statistical difference between the groups (Table 3).

**Table 3:** Descriptive blood gas measurements in the animals that completed the study protocol.

Variable	Time	Sham group	CG group	GAT1 group	*p* level
N	Basal	4	38	16	
	10 min ROSC	–	16	10	
	4 h ROSC	4	10	10	
Temperature(°C)	Basal	35.7 ± 0.2	35.5 ± 0.8	35.5 ± 0.9	0.985
	10 min ROSC	35.6 ± 0.3	34.7 ± 1.02	35.1 ± 1.3	0.690
	4 h ROSC	36.1 ± 0.2	36.5 ± 0.4	35.8 ± 0.9	0.084
pH	Basal	7.36 ± 0.07	7.42 ± 0.06	7.41 ± 0.04	0.725
	10 min ROSC	–	7.060 ± 0.186	7.170 ± 0.103	0.032
	4 h ROSC	7.310 ± 0.070	7.268 ± 0.220	7.358 ± 0.101	0.726
pCO_2_(mmHg)	Basal	34.4 ± 10.2	32.6 ± 4.0	29.4 ± 4.4	0.093
	10 min ROSC	–	43.9 ± 45.7	45.4 ± 8.5	0.930
	4 h ROSC	47.8 ± 11.6	32.6 ± 16.0	33.7 ± 11.4	0.153
pO_2_ (mmHg)	Basal	330.2 ± 22.6	218.5 ± 92.6	275.5 ± 44.9	0.242
	10 min ROSC	–	201.2 ± 100.3	182.4 ± 81.8	0.630
	4 h ROSC	271.5 ± 27.3	325.7 ± 62.2	273.0 ± 107.9	0.285
Na (mmol/L)	Basal	144.5 ± 2.1	138.6 ± 4.4	139.5 ± 3.81	0.453
	10 min ROSC	–	139.3 ± 7.7	143.3 ± 8.5	0.100
	4 h ROSC	145.0 ± 3.4	141.0 ± 5.9	144.4 ± 3.6	0.138
K (mmol/L)	Basal	4.0 ± 0.3	4.1 ± 0.6	3.6 ± 0.4	0,130
	10 min ROSC	–	3.6 ± 3.4	3.1 ± 0.5	0.045
	4 h ROSC	4.7 ± 0.7	4.6 ± 1.2	3.9 ± 0.6	0.180
Cl (mmol/L)	Basal	110.1 ± 2.3	111.1 ± 2.9	110.1 ± 4.3	0.631
	10 min ROSC	–	109.4 ± 4.4	110.6 ± 4.1	0.413
	4 h ROSC	108.2 ± 7.1	113.5 ± 7.38	113.6 ± 3.6	0.372
Ca (mmol/L)	Basal	0.86 ± 0.21	0.91 ± 0.18	0.80 ± 0.19	0.216
	10 min ROSC	–	0.89 ± 0.19	0.75 ± 0.18	0.037
	4 h ROSC	0.97 ± 0.34	0.89 ± 0.22	0.71 ± 0.08	0.112
Glucose (mg/dL)	Basal	314.0 ± 85.6	295.5 ± 75.5	260.0 ± 65.1	0.346
	10 min ROSC	–	441.2 ± 106.8	393.0 ± 73.6	0.109
	4 h ROSC	230 ± 139	234 ± 80	233 ± 88	0.832
Lactate (mmol/L)	Basal	2.50 ± 0.81	2.79 ± 0.95	3.15 ± 1.10	0.320
	10 min ROSC	–	10.64 ± 5.93	7.79 ± 3.87	0.257
	4 h ROSC	2.92 ± 1.73	5.78 ± 3.89	2.93 ± 1.60	0.341
Hb (g/dL)	Basal	16.1 ± 2.35	16.7 ± 0.92	16.1 ± 1.31	0.356
	10 min ROSC	–	16.6 ± 1.53	15.8 ± 1.26	0.215
	4 h ROSC	15.85 ± 1.83	17.4 ± 2.05	16.9 ± 1.34	0.350
Ht (%)	Basal	46.3 ± 4.04	49.2 ± 5.85	49.2 ± 3.97	0.235
	10 min ROSC	–	51.3 ± 5.3	48.6 ± 3.8	0.206
	4 h ROSC	45.8 ± 3.2	53.1 ± 6.1	51.9 ± 4.0	0.037
BE (mmol/L)	Basal	− 2.87 ± 1.2	− 4.11 ± 1.6	− 2.63 ± 1.5	0.406
	10 min ROSC	–	− 14.9 ± 8.0	− 10.6 ± 4.9	0.048
	4 h ROSC	-2.5 ± 3.7	− 9.1 ± 10.4	− 5.9 ± 5.3	0.342
Bic (mmol/L)	Basal	21.6 ± 2.3	21.3 ± 1.5	19.6 ± 1.6	0.056
	10 min ROSC	–	13.5 ± 6.3	16.6 ± 3.7	0.037
	4 h ROSC	23.2 ± 3.7	16.7 ± 8.2	18.7 ± 4.8	0.180

Descriptive measures of arterial blood gas baseline parameters after the return of spontaneous circulation and at the end of the study. Kruskall-wallis test. BE: base excess; Bic: bicarbonate.

### Cerebral and myocardial injury

**Figure 5:**
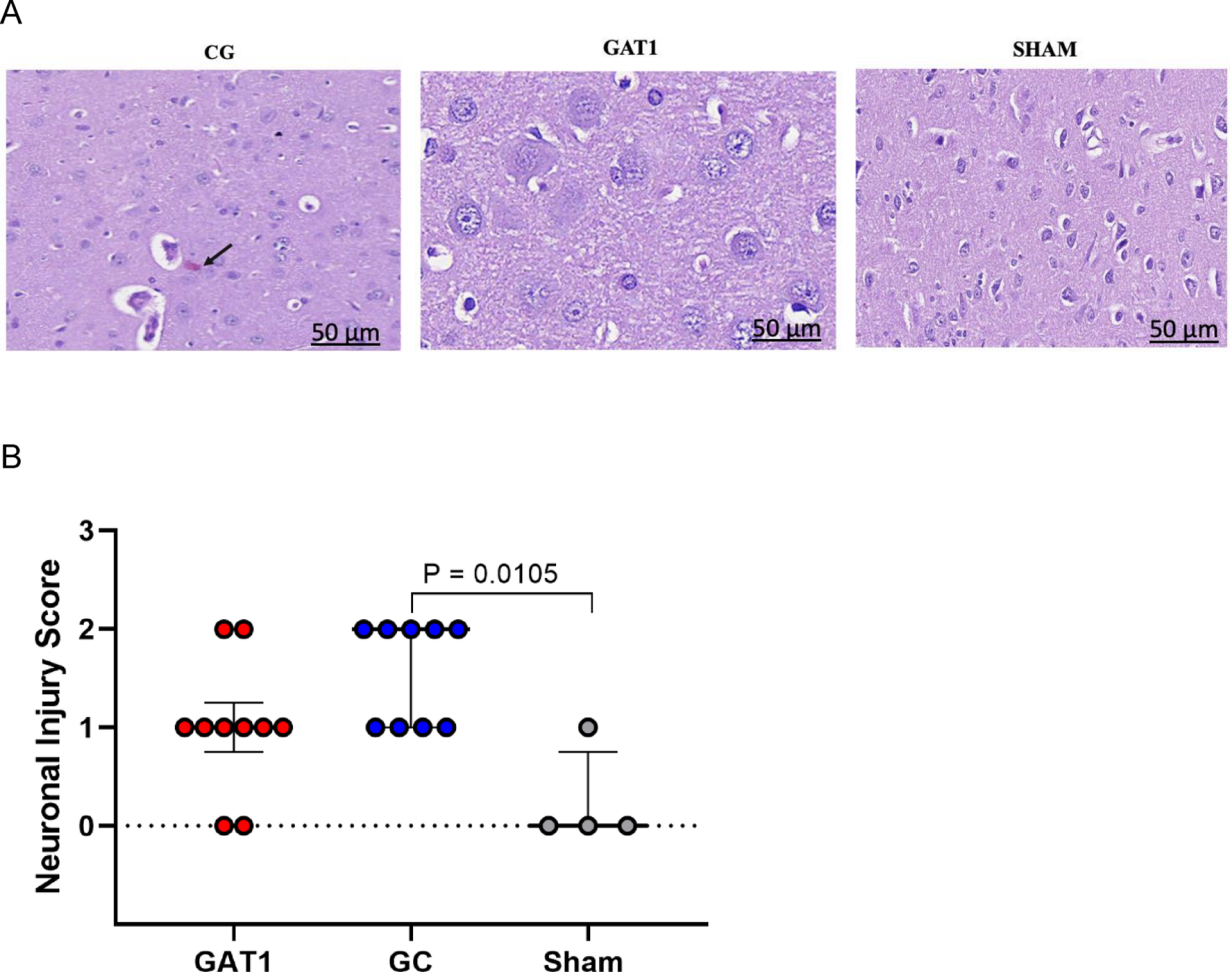
(**A**) and (**B**) Histological analysis of cerebral tissue samples collected from rats subjected to cardiac arrest and treated with adrenaline (GC, n = 10) or candesartan (GAT1, n = 10) and sham-operated rats (Sham, n = 4). (**A**) Cerebral tissue sections stained with H&E, showing neuronal injury at 4 h after sepsis induction. Magnification 50 μm × 100. The black arrow represents red neurons. (**B**) Neuronal injury score measured in the brain cortex.

On cerebral evaluation, microscopic evaluation showed more red neurons and signs of necrosis throughout the parietal cortex and hippocampus in the CG group compared to the GAT1 group (*p* = 0.025). The Sham group showed no signs of neuronal injury (Fig. 5A). The distribution of neuronal injury scores between the groups is seen in Fig. 5B.

**Figure 6:**
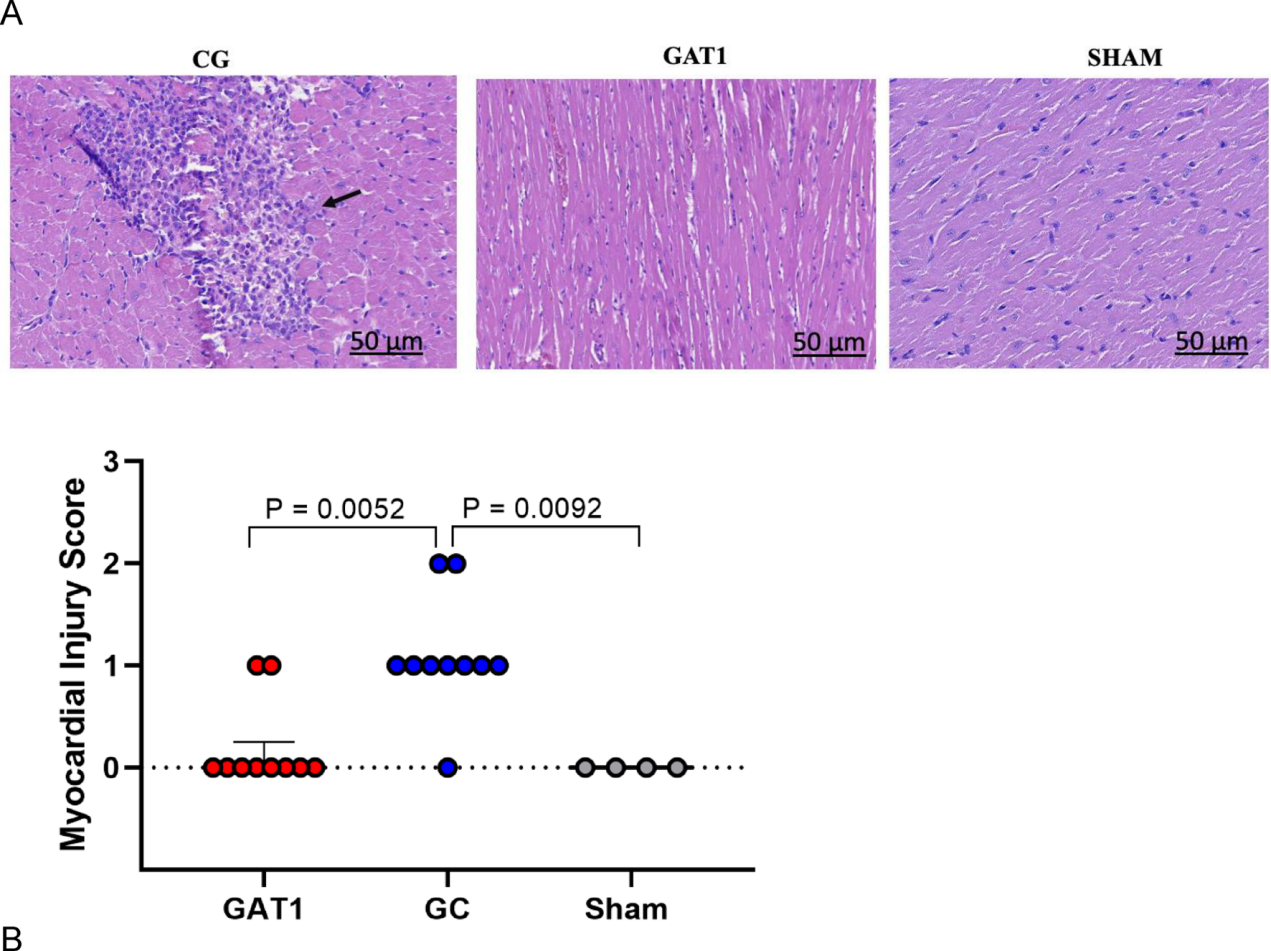
(**A**) and (**B**). Histological analysis of myocardial tissue samples collected from rats subjected to cardiac arrest and treated with adrenaline (GC, n = 10) or candesartan (GAT1, n = 10) and sham-operated rats (Sham, n = 4). (**A**) Heart tissue sections stained with H&E, showing myocardial injury at 4 h after cardiac arrest induction. Magnification 50 μm × 100. The black arrow represents lymphocytic infiltrate. (**B**) Myocardial injury score measured in the subendocardial layer in the left and right ventricle.

On microscopic evaluation, myocardial cells were orderly arranged with intact nuclei in the Sham group. In the CG group, myocardial cells were irregular, and many immune cells were aggregated (Fig. 6A). Compared to the GAT1 group, fewer myocardial cells were disorganized, and cell infiltrations were lower in the latter group (Fig. 6B). The CG's myocardial damage score increased significantly compared with the Sham group. Furthermore, the myocardial damage score significantly decreased in the GAT1 group compared to the CG group, as seen in Fig. 6B.

When using the TUNEL method, similar medians were observed for the number of apoptotic cells in both brain and heart groups. The Sham group showed no signs of cell injury.

## Discussion

To the best of our knowledge, this is the first report that evaluates the effect of AT1 receptor blockade during cardiac arrest as an adjunct to advanced life support. We demonstrated that the antagonism of Angiotensin II AT1 receptor with candesartan leads to a higher rate of ROSC and survival in rats after CA. The results also indicate that candesartan attenuates neuronal and myocardial injury when administered during CPR maneuvers.

Effective cardioprotective strategies after ROSC are scarce in the literature. Therefore, finding interventions that effectively improve post-resuscitation myocardial and neuronal dysfunction is critical for patient prognosis.

Cardiac dysfunction associated with post-CPR syndrome is characterized by severe myocardial impairment and global hypokinesia, affecting the success rate of cardiopulmonary resuscitation. Cardiac function deterioration begins minutes after arrest and peaks within 2 to 5 h after resuscitation, reinforcing early intervention and treatment^32^. Proposed molecular mechanisms for post-resuscitation myocardial dysfunction involve I/R injury, resulting in large amounts of oxygen free radicals that damage cell membranes and induce myocyte necrosis and apoptosis. Moreover, an intracellular accumulation of Na + occurs through the cytosolic overload of Ca^2+^ under the action of the Na^+^/Ca^2+^ exchanger present in the sarcolemma membrane^33^.

The presence of endogenous or paracrine RAS in the heart has been recently recognized. RAS components—angiotensinogen and renin messenger RNA, angiotensin I to angiotensin II (Ang II) converting enzyme—have previously been detected in the heart and seem functionally integrated^11–14^. Cardiac Ang II may be involved in regulating coronary blood flow, modulation of sympathetic neurotransmission, cardiac contractility, and stimulation of cell hyperplasia and hypertrophy, in addition to repairing the cardiovascular structure. Angiotensin II interacts with two pharmacologically distinct subtypes of cell surface receptors, type 1 and type 2 Ang II receptors. Type 1 receptors mediate the main cardiovascular effects of Ang II^25^. Such mechanisms may explain the significant differences found in the lower myocardial injury score in the GAT1 group reported here.

Furthermore, limiting Na^+^ influx into the sarcolemma during ventricular fibrillation resuscitation prevents the accumulation of excess mitochondrial Ca^2+^ and attenuates myocardial injury^34^. This mechanism may explain the significant reduction in cardiac arrhythmias observed here since no differences in CPR duration, number of defibrillations, or adrenaline doses between groups were observed. Thus, we suggest that the action of candesartan does not interfere with the reversal of heart rhythm (VF or VT) during CPR. However, as the incidence of cardiac arrhythmias one hour after ROSC was higher in the control group, the drug could potentially affect the reduction of new arrhythmogenic triggers. This mechanism leads to the blockade of Angiotensin II AT1 receptors, explaining the higher survival of the animals in the GAT1 group, as demonstrated in previous studies^22–25^.

Since retrospective studies have suggested that high cumulative epinephrine dosage is associated with worse hemodynamic and neurological outcomes and, although it may improve coronary perfusion and increase vascular resistance to promote initial ROSC during CPR, these same effects may lead to increased myocardial dysfunction and occasionally a severe toxic hyperadrenergic state in the post-resuscitation period^35–39^.

Based on the facts, previous animal studies have shown that using large doses of sodium nitroprusside during CPR significantly improved hemodynamics when used as a simulated ACLS intervention^40^. Our hypothesis is that ARA could behave similarly, optimizing vital organ perfusion pressures and redirecting blood flow to the brain and thorax, significantly improving myocardial perfusion, and facilitating spontaneous ROSC.

The findings from brain histopathology point to candesartan as a potential neuroprotector in ischemic injury caused by CA. Hyperactivation of the brain AT1 receptor is responsible for the harmful effects associated with the RAS, leading to vasoconstriction, cerebral blood flow decrease, increased oxidative stress, and vulnerability to ischemia, in addition to promoting vascular and tissue inflammation and neurodegeneration exacerbation^41^. AT2 stimulation counteracts these mechanisms. Therefore, selective blocking of AT1 receptors with Angiotensin Block Receptors (ABRs), especially candesartan, which crosses the blood–brain barrier^42^, may offer superior protection than simultaneously decreasing AT1 and AT2 receptors, such as angiotensin-converting enzyme inhibitors (ACE inhibitors). Such mechanisms were used by Hajjar et al. (2020)^43^, demonstrating less neurocognitive impairment in an elderly population that used candesartan instead of Lisinopril, regardless of blood pressure control.

Recently, AT2 receptors have also been linked to neuroprotection, especially after a stroke. These studies have shown that under pathological conditions, such as ischemic insult, AT2 receptor expression may be upregulated and it has been speculated that the increased stimulation of the AT2 receptor may be responsible for some of the therapeutic effects observed during AT1 receptor antagonism^44, 45^.

It has also been shown that the coadministration of an AT2 receptor antagonist decreases the potential neuroprotection conferred by candesartan or irbesartan on animal models of cerebral artery occlusion, clearly demonstrating that AT2 receptor activation contributed to the effect of AT1 receptor antagonists^44, 46^. This is also supported by the fact that AT2 receptor-deficient mice have larger infarct volumes and poorer neurological outcomes after stroke^45, 47^.

Our study lacks a group with AT2 receptor antagonism, making it unclear whether the neuro- and cardioprotective effects were due only to the AT1 receptor antagonism or could be from AT2 stimulation. This is a limitation of our study and further investigations are needed to clear this question.

As for the metabolic analysis of I/R, the GAT1 group had a lower level of acidosis and base intake than the CG group, suggesting that the combination of adrenaline and candesartan can protect myocardial tissue and improve energy metabolism after ROSC. Since candesartan is one of the most potent aldosterone suppressors and higher potassium levels are implicated in lower ROSC rates, we expected higher potassium levels on GAT1^48–50^. However, potassium levels were lower 10 min after ROSC. We infer that these findings might be attributed to the higher pH of this group. In addition, aldosterone has been shown to induce myocyte apoptosis in the heart, which candesartan might have blocked, also explaining the better cardioprotection in this group^51^.

The best survival rate for the GAT1 group corroborates this hypothesis. Furthermore, the lower blood pressure in the GAT1 group compared to the control and Sham groups may be due to the hypotensive effect of angiotensin II antagonism^10^. This finding may compromise the translation of this experimental study into clinical studies since we do not know the behavior of this drug after CPR in humans.

### Limitations

The main limitations of this study include the absence of a group that received candesartan alone during resuscitation maneuvers. However, the use of adrenaline as a significant factor for ROSC increase in rats has already been documented^15^.

Another limitation of this study is that we have only evaluated the animals for a short period (4 h); thus, long-term effects could not be investigated. In addition, we have not tested other doses of candesartan infusion, which could limit the side effects and still have the potential for organ protection. Since the ROSC rates were lower than the experimental model adopted by our research group^27^, more animals were required to reach 10 per intervention and control group, as indicated in the study design. Although based on estimated sample calculation, the small sample size may reduce the study's statistical power. Our study is primarily descriptive, and exploratory mechanisms are needed to better clarify our findings.

Our results suggest good perspectives for using angiotensin II AT1 receptor antagonists for cardiopulmonary resuscitation protocols, especially in assisted arrests, where the intervention time is shorter compared to out-of-hospital CAs.

## Data Availability

Availability of data upon request to the main author Araujo Filho EAF (elsonfilho2@hotmail.com).
